# MiCK: a database of gut microbial genes linked with chemoresistance in cancer patients

**DOI:** 10.1093/database/baae124

**Published:** 2024-12-21

**Authors:** Muhammad Shahzaib, Muhammad Muaz, Muhammad Hasnain Zubair, Masood Ur Rehman Kayani

**Affiliations:** Metagenomics Discovery Lab, School of Interdisciplinary Engineering and Sciences (SINES), National University of Sciences and Technology (NUST), Islamabad 44000, Pakistan; School of Electrical Engineering and Computer Science (SEECS), National University of Sciences and Technology (NUST), Islamabad 44000, Pakistan; Metagenomics Discovery Lab, School of Interdisciplinary Engineering and Sciences (SINES), National University of Sciences and Technology (NUST), Islamabad 44000, Pakistan; Metagenomics Discovery Lab, School of Interdisciplinary Engineering and Sciences (SINES), National University of Sciences and Technology (NUST), Islamabad 44000, Pakistan

## Abstract

Cancer remains a global health challenge, with significant morbidity and mortality rates. In 2020, cancer caused nearly 10 million deaths, making it the second leading cause of death worldwide. The emergence of chemoresistance has become a major hurdle in successfully treating cancer patients. Recently, human gut microbes have been recognized for their role in modulating drug efficacy through their metabolites, ultimately leading to chemoresistance. The currently available databases are limited to knowledge regarding the interactions between gut microbiome and drugs. However, a database containing the human gut microbial gene sequences, and their effect on the efficacy of chemotherapy for cancer patients has not yet been developed. To address this challenge, we present the Microbial Chemoresistance Knowledgebase (MiCK), a comprehensive database that catalogs microbial gene sequences associated with chemoresistance. MiCK contains 1.6 million sequences of 29 gene types linked to chemoresistance and drug metabolism, curated manually from recent literature and sequence databases. The database can support downstream analysis as it provides a user-friendly web interface for sequence search and download functionalities. MiCK aims to facilitate the understanding and mitigation of chemoresistance in cancers by serving as a valuable resource for researchers.

**Database URL**: https://microbialchemreskb.com/

## Introduction

Cancer is a significant global health concern, ranking among the leading causes of morbidity and mortality worldwide. According to the WHO, cancer accounted for nearly 10 million deaths in 2020 and was ranked as the second leading cause of global deaths. The GLOBOCAN report highlights that there were approximately 19.3 million new cancer cases and 10 million cancer-related deaths in 2020 [1]. Factors contributing to the incidence of cancer include genetic predisposition, lifestyle factors such as diet, obesity, and alcohol consumption, as well as environmental exposures and infections. Multiple treatment strategies are available for cancer, ranging from surgical interventions to chemotherapy, radiation therapy, immunotherapy, and targeted therapies. The approach to treatment often depends on the type and stage of cancer, as well as the patient’s overall health and preferences [2].

Major drugs used to treat cancer include 5-Flurouracil (5-FU), Capecitabine, Gemcitabine, Irinotecan, and Oxaliplatin. 5-FU is a commonly used antimetabolite drug for cancer that causes the inhibition of thymidylate synthase, an enzyme essential for DNA synthesis and repair. Capecitabine, a prodrug of 5-FU, is converted to 5-FU in tumor tissues by the enzyme thymidine phosphorylase [3]. Furthermore, it has replaced 5-FU due to its improved pharmacokinetics and tolerability [4]. Gemcitabine is a nucleoside analog that inhibits DNA synthesis by interfering with the activity of the ribonucleotide reductase enzyme [5]. Irinotecan, a DNA topoisomerase inhibitor, binds to topoisomerase which causes the inhibition of DNA replication and transcription through its active metabolite i.e. SN38 [3]. Oxaliplatin works by forming DNA adducts inhibiting DNA replication, transcription, DNA damage, and apoptosis of cancer cells [3].

Chemoresistance is a major challenge in the successful treatment of cancer patients. The human gut microbiome has recently been attributed to the emergence of chemoresistance in cancer patients [6]. Gut microbial species including *Escherichia coli*, *Bifidobacterium longum, Citrobacter*, and *Enterococcus faecalis* metabolize 5-FU, the most common drug used in treating cancer patients, and alter its efficacy [7]. Another prominent example includes the increased efflux of oxaliplatin by gut microbes through the glucuronidation mechanism by the β-glucuronidase enzyme. This enzyme inactivates SN38 (an activated form of irinotecan) by converting it to SN38G [8]. *Fusobacterium nucleatum*, another common gut microbial species, promotes chemoresistance by the modulation of autophagy [9]. *Lactobacillus* and *Bifidobacterium* have been found to have higher abundance in the progressive disease group compared to the partial response group in cases administered with folinic acid, 5-FU, and irinotecan [10].

Several databases have been designed, considering the importance of the gut microbiome and its interaction with drugs used in chemotherapy. These include PharmacoMicrobiomics which contains 131 records about the effect of drugs on gut microbiome [6]. Microbe-Drug Association Database contains interactions of 1388 drugs, and 180 microbes curated from drug databases and relevant literature [9]. MagMD is also a comprehensive database for metabolic actions of the gut microbiome on drugs, covering 32 678 interactions between 2146 microbes, 36 enzymes, and 219 substrates [11]. To the best of our knowledge, no database currently exists that provides dedicated access to microbial gene sequences potentially involved in chemoresistance, which can support downstream analysis for therapeutic interventions. To address this challenge, we present the Microbial Chemoresistance Knowledgebase (MiCK), a comprehensive database cataloging microbial gene sequences associated with chemoresistance in CRC. MiCK contains 1.6 million sequences of 29 gene types linked to chemoresistance and drug metabolism, curated from recent literature and databases. The database architecture, built using MySQL, supports efficient data search, retrieval, and download functionalities through its user-friendly web interface. MiCK is designed to facilitate the understanding and mitigation of chemoresistance in cancer treatment, serving as an invaluable resource for researchers.

## Materials and methods

### Identification of gut microbial chemoresistance genes

For the identification of gut microbial genes, potentially associated with chemoresistance, we first performed a literature search in PubMed [8] and Google Scholar [7] using the following keywords: “chemoresistance,” “cancer,” and “gut microbiome.” 16S rRNA-based studies were excluded. Through manual curation, the search results were restricted to only the publications that reported or discussed microbial genes or products that could potentially result in chemoresistance. In addition, enzymes involved in the metabolism of commonly used therapeutics for cancer were searched in the KEGG [12] and MagMD databases [11].

After collecting information on genes and enzymes, we retrieved their sequences from the UniProtKB, and NCBI Gene databases [13]. Full gene name was used for searching sequences in the databases, from the search results, only bacterial sequences were downloaded for downstream analysis. Through this, we identified 29 gene types that could potentially confer chemoresistance. The genes targeting gemcitabine included cytidine deaminase, thymidine kinases (*TK*), deoxycytidine kinase (*dck*), histone deacetylase (*HDAC*), and ribonucleotide reductase (*RNR*). Cytidine deaminase, produced by *Gammaproteobacteria*, is known to inactivate Gemcitabine [14]. *TK* are involved in the salvage pathway capable of synthesizing deoxythymidine by phosphorylation of thymidine thus helping in DNA synthesis, whereas knocking *TK2* by small interfering RNA sensitizes the cell to gemcitabine chemotherapy, thus confirming their role in chemoresistance [15]. *dck* is reported to be involved in limiting the cytotoxic activity of gemcitabine [16]. *HDAC6* is reported to be upregulated in colorectal cancer patients and thus plays a role in poor prognosis and survival [17]. *RNR* consists of two subunits M1 and M2. M1 subunit has been identified in large amounts in microarray profiles of *in vivo* gemcitabine resistance model, which depicts its role in chemoresistance [18].

The target genes for oxaliplatin include butyrate, cysteine desulfurase (*NFS1*), metallothionein (*MT*), glutathione (*GLU*), and glutathione S-transferase (*GST*). Butyrate, a short-chain fatty acid produced by gut microbes, has been reported to play a dual role. Its high concentration in oxaliplatin-resistant cell lines can be considered evidence of its role in chemoresistance [19]. *NFS1* weakens the sensitivity of cancer cells to oxaliplatin [20], whereas *MT* are small cystine-enriched molecules with four isoforms, *MT1* to *MT4*. Thiol groups of *MT* are involved in chemoresistance to oxaliplatin, a drug used to treat CRC through drug detoxification, which prevents cells from apoptosis and accumulation of oxaliplatin [21]. *GLU* conjugates with oxaliplatin, and this conjugate serves as the substrate for detoxification enzymes [22], whereas *GST* is a detoxification enzyme for oxaliplatin [23].

Target genes of 5-FU included dihydropyrimidine dehydrogenase (*DHP*), dutpase, nucleoside diphosphate kinase (*NME2*), thymidylate synthase (*TS*), toll-like receptors (TLRs), UMP/CMP kinase, and thymidine phosphorylase. *DHP* causes catabolism of 5-FU [24], whereas the expression of Dutpase is linked to susceptibility of cancer cells to chemotherapeutics like 5-FU [25]. Knockdown of the *NME2* gene increases sensitivity to 5-FU in CRC cell lines [26], whereas 5-FU inhibits the TS, which stops DNA replication and apoptosis of tumor cells. One of the key indicators of chemoresistance to 5-FU is an increase in the concentration of TS in tumor cells [22]. TLR is the main receptor in the pathway that can mitigate the bacterial production of pro-inflammatory cytokines that help in tumorigenesis and can lead to chemoresistance in cancer cells [27]. An active TLR-4-MyD88 signaling pathway could pose a risk for cancer development and serve as a promising target for creating biomodulators to combat chemoresistance [28]. UMP/CMP kinase attenuates the functioning of 5-FU and leads to chemoresistance against it [29]. Thymidine phosphorylase is involved in the activation of 5-FU, but it is also involved in tumor progression through angiogenesis and by avoiding apoptosis, which depicts its role in chemoresistance against 5-FU-based chemotherapy of cancer [30].

Target genes for irinotecan included UDP-glucuronosyltransferases (*UGTs*), β-glucuronidase (*BGU*), topoisomerase inhibitors (*DTI*), and Hydroxyglutarate. *UGTs* impair the biological activity by glucuronidation and enhancing the water solubility of the drugs [31]. UMP/CMP kinase is an enzyme involved in the phosphorylation of 5-FU, but miR-130-b which is a key epigenetic regulator of *BGU*, an enzyme produced mostly by four gut bacterial phyla, namely *Bacteroidetes, Firmicutes, Verrucomicrobia*, and *Proteobacteria*. *BGU* induces toxicity in response to irinotecan thus limiting the dose and affecting the efficacy of irinotecan [32]. Most of the chemotherapeutics are topoisomerase-active substances but the *DTIs* are involved in the chemoresistance against these drugs. DTIs include ATP-binding cassette transporters (*ABC*), *GSH*, and *HDAC6* [33]. Hydroxyglutarate has been shown to inhibit α-ketoglutarate-dependent dioxygenases, including histone demethylases, which leads to epigenetic changes that can promote tumor progression and chemotherapeutic resistance [34].

The gut microbiome can influence the functionality of drugs by secreting several enzymes [27]. Therefore, to enhance the pool of genes that can potentially result in chemoresistance, we searched for pathways of drug metabolism in the KEGG Pathways [12] and explored the MagMD database that provided the information about interaction of microbial enzymes and drugs [11]. We then retrieved the sequences of all the enzymes from UniProtKB and NCBI gene databases. β-ureidopropionase, phosphoribosyl transferase, uridine phosphorylase, uracil phosphoribosyl transferase, carboxyl esterase, uridine kinase, and deoxycytidine kinase were the enzymes involved in the metabolism of cancer drugs and were used in our database. The list of chemoresistance genes (CRGs) is provided in Table 1.

**Table 1.** Chemoresistance genes, target drugs, and the potential effects of the genes

CRGs	Drug	Effect	Reference
Butyrate	Oxaliplatin	Chemoresistance	[19]
Cytidine deaminase	Gemcitabine	Chemoresistance	[14]
Cystine desulfurase	Oxaliplatin	Decreased sensitivity/ Chemoresistance	[20]
Dihydropyridine dehydrogenase	Capecitabine/5-FU	Catabolism	[24]
Glutathione	Oxaliplatin	Chemoresistance	[35]
Glutathione s transferase	Oxaliplatin	Detoxification enzyme	[23]
UDP-glucuronosyltransferases	Irinotecan	Chemoresistance	[31]
Nucleoside diphosphate kinase	Capecitabine	Chemoresistance	[26]
Ribonucleotide reductase	Gemcitabine	Chemoresistance	[18]
Thymidine kinases	Gemcitabine	Chemoresistance	[15]
Thymidylate synthase	5-FU	Drug target	[22]
UMP/CMP kinase	Capecitabine	Chemoresistance	[29]
β-glucuronidase	Irinotecan	Reduced efficacy	[32]
ATP-binding cassette transporters	Oxaliplatin	Efflux of drugs	[33]
Metallothionein	Oxaliplatin	Chemoresistance	[21]
Hydroxyglutarate	Gemcitabine	Chemoresistance	[34]
DNA topoisomerase inhibitors	Irinotecan	Chemoresistance	[33]
Carboxyl esterase	Capecitabine	Substrate metabolism	[11]
dUTPase	Capecitabine	Chemoresistance	[36]
Histone deacetylase	Gemcitabine	Chemoresistance	[17]
Purine nucleoside phosphorylase	5-FU	Chemoresistance	[26]
Thymidine phosphorylase	Capecitabine	Chemoresistance	[30]
Toll like receptors	5-FU	Chemoresistance	[28]
β-ureidopropionase	Capecitabine	Substrate metabolism	[11]
Phosphoribosyl transferase	5-FU	Substrate metabolism	[11]
Uridine phosphorylase	Capecitabine	Substrate metabolism	[11]
Uracil phosphoribosyl transferase	Capecitabine	Substrate metabolism	[11]
Uridine kinase	5-FU	Substrate metabolism	[11]
Deoxycytidine kinase	Gemcitabine	Substrate metabolism	[11]

### Non-redundant CRG catalogue and database construction

MMseqs2 (version 13.45111) was used to cluster the CRGs [37]. MMseqs2 was used for indexing the sequence database (generated from the concatenation of all sequences), followed by clustering using linclust. Clustering was performed using multiple identity thresholds (i.e. 50%, 75%, 80%, 90%, and 100%) with query coverage of 80%.

**Figure 1.**
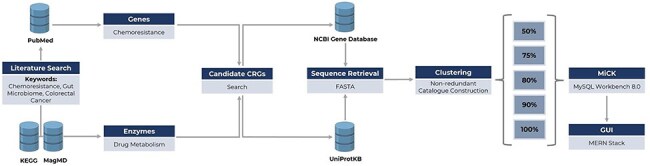
Framework of MiCK development, including (i) literature search for the identification of genes/enzymes linked to chemoresistance, (ii) retrieval of respective gene and enzyme sequences from NCBI Gene and UniProtKB databases, and (iii) sequence clustering for constructing MiCK using MySQL Workbench.

The database was created using MySQL (version 8.0.2, https://www.mysql.com/) and consists of five columns: “Accession_number,” “Gene_type,” “Drug,” “Uniport_Accession,” and “Effects.” The accession number serves as the primary key of the database, the second column contains gene type information, the third column provides the target drug for the gene type, the fourth column holds the full gene type names, the fifth column contains the original accession numbers for retrieving sequences from UniProtKB, and the last column holds the information of effects on the drugs. The framework of the database construction is illustrated in Fig. 1.

### Graphical user interface design

The MiCK interface was developed using the MERN stack, integrating MongoDB as the database, Express.js for the backend, React.js for the front end, and Node.js as the server environment. MongoDB stores data in a flexible, JSON-like format, while Express.js establishes API endpoints for data retrieval and management, ensuring secure communication between the user and the database. React.js builds a responsive interface using reusable components and state management. Node.js handles server-side operations, processing concurrent requests efficiently. The platform’s components are connected through RESTful architecture, with React sending HTTP requests to the Express backend, which interacts with MongoDB to maintain a user-friendly experience.

### MiCK statistics

**Figure 2.**
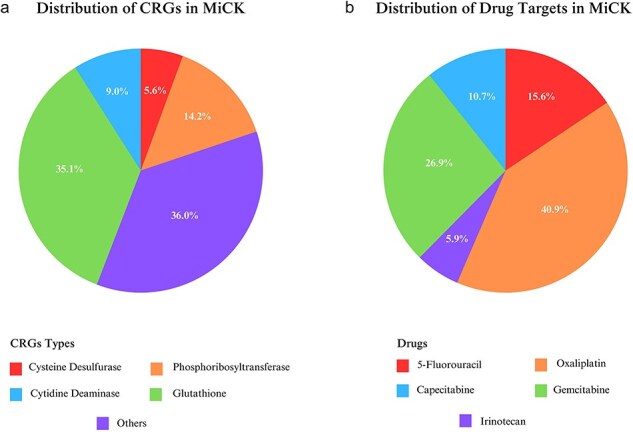
Statistics of distribution of chemoresistance genes (a) and their target drugs (b) included in MiCK.

MiCK hosts ∼1.6 million sequences that encompass 29 CRGs, obtained through a literature search and an extensive review of drug-metabolizing enzymes. Among these, *GLU* is the most frequent gene type included in MiCK with ∼0.6 million representative gene sequences, followed by *PRT* (∼0.25 million gene sequences) (Fig. 2a). MiCK contains ∼0.6 million genes that target oxaliplatin, followed by gemcitabine for which ∼0.4 million target gene sequences are present in MiCK (Fig. 2b). The effects column comprises chemoresistance, chemosensitivity, and substrate metabolism. Chemoresistance is the most frequent effect with almost 1.3 million repetitions.

### Database web interface

MiCK offers a user-friendly interface with six navigation bars, i.e. Home, Statistics, Search, Downloads, Team, and About.

#### Home

The home page welcomes MiCK users to the website and also provides a basic description of cancer and problems associated with chemoresistance, and the necessity to create MiCK. From this page, the users can navigate to various other pages of the website, which include Statistics, Search, Downloads, Teams, and About pages.

#### Statistics

The statistics page of the MiCK features interactive pie charts that display statistics for chemoresistance genes and drug targets in MiCK. The users can click on the categories, and they will be brought to the search page showing results corresponding to the clicked category. This feature enhances user experience and makes data exploration more efficient.

#### Search

The search page comprises a selection and search boxes. Through the selection box, the user can select the search criteria, whereas the search box allows keyword input. Users can search through accession numbers, drug names, gene names, and effects. The resultant table displays information on gene type, its drug target, potential effect on the drug, and the accession number of sequences.

#### Downloads

**Figure 3.**
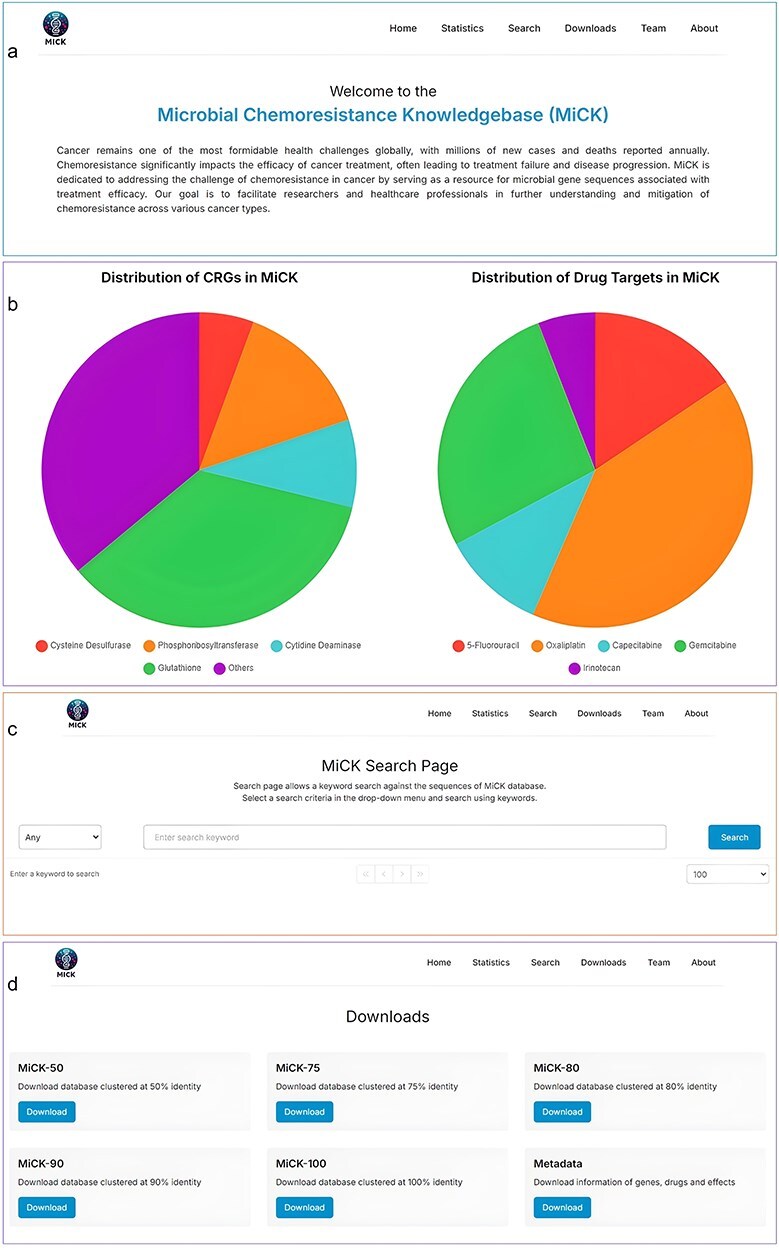
Major components of the MiCK graphical user interface: (a) The Home page, (b) The Statistics page, (c) The Search page, and (d) The Downloads page.

The downloads page offers the utility to download the CRG catalog, which includes the sequencing files as well as the metadata. Currently, users can download the CRG catalog clustered at 50%, 75%, 80%, 90%, and 100% similarity cutoffs. Metadata includes information about the CRGs, corresponding drugs, and effects on the drug. The graphical user interface of MiCK is shown in Fig. 3.

#### Team

The Team page provides information and contact details for the Principal Investigator, author, co-author, and database developer. This section ensures transparency and facilitates direct communication with the key contributors to the MiCK project.

#### About

The About page provides help for the users by answering some of the most frequently asked questions that a potential user can have in mind. In addition, the contact information of the database administrator is also provided for further queries.

## Data Availability

The publicly retrieved gene sequences and relevant details can be downloaded from Zenodo using doi: https://doi.org/10.5281/zenodo.14190584.

## References

[1] WHO. Cancer Today. 2022. https://gco.iarc.fr/today/en (3 April 2024, date last accessed).

[2] WHO. Cancer. 2022. https://www.who.int/news-room/fact-sheets/detail/cancer (2 July 2024, date last accessed).

[3] Grivicich I , Mans DRA, Peters GJ et al. Irinotecan and oxaliplatin: an overview of the novel chemotherapeutic options for the treatment of advanced colorectal cancer. *Braz J Med Biol Res* 2001;34:1087–103. doi: 10.1590/S0100-879X2001000900001 11514832

[4] Elgemeie GH , Mohamed-Ezzat RA. *Pyrimidine-based Anticancer Drugs, New Strategies Targeting Cancer Metabolism*1st edn. Radarweg 29, 1043 NX Amsterdam, The Netherlands: Elsevier, 2022, 107–42.

[5] Zoetemelk M , Ramzy GM, Rausch M et al. Drug-drug interactions of irinotecan, 5-Fluorouracil, folinic acid and oxaliplatin and its activity in colorectal carcinoma treatment. *Molecules* 2020;25:2614. doi: 10.3390/MOLECULES25112614 PMC7321123 32512790

[6] Elrakaiby M , Dutilh BE, Rizkallah MR et al. Pharmacomicrobiomics: the impact of human microbiome variations on systems pharmacology and personalized therapeutics. *OMICS* 2014;18:402–14. doi: 10.1089/OMI.2014.0018 24785449 PMC4086029

[7] Zhang W , Song H, Yang S et al. Deciphering the Antibacterial Mechanisms of 5-Fluorouracil in Escherichia coli through Biochemical and Transcriptomic Analyses. *Antibiotics* 2024;13:528. doi: 10.3390/antibiotics13060528 PMC11200800 38927194

[8] Takakura A , Kurita A, Asahara T et al. Rapid deconjugation of SN-38 glucuronide and adsorption of released free SN-38 by intestinal microorganisms in rat. *Oncol Lett* 2012;3:520–4. doi: 10.3892/ol.2011.519 22740943 PMC3362476

[9] Sun YZ , Zhang DH, Bin Cai S et al. MDAD: a special resource for microbe-drug associations. *Front Cell Infect Microbiol* 2018;8:424. doi: 10.3389/FCIMB.2018.00424 PMC6292923 30581775

[10] Chen YC , Chuang CH, Miao ZF et al. Gut microbiota composition in chemotherapy and targeted therapy of patients with metastatic colorectal cancer. *Front Oncol* 2022;12:955313. doi: 10.3389/FONC.2022.955313 PMC9539537 36212420

[11] Zhou J , Ouyang J, Gao Z et al. MagMD: database summarizing the metabolic action of gut microbiota to drugs. *Comput Struct Biotechnol J* 2022;20:6427. doi: 10.1016/J.CSBJ.2022.11.021 PMC9685347 36467581

[12] Kanehisa M . The KEGG Database. Gregory B., Jamie AG. *In silico’simulation of biological processes: Novartis Foundation Symposium* Chichester, UK: John Wiley & Sons, Ltd, 2002;247.

[13] The UniProt Consortium . UniProt: a hub for protein information. *Nucleic Acids Research* 2015;43:D204–12. doi: 10.1093/nar/gku989 25348405 PMC4384041

[14] Qi X , Liu Y, Hussein S et al. The species of gut bacteria associated with antitumor immunity in cancer therapy. *Cells* 2022;11:3684. doi: 10.3390/CELLS11223684 PMC9688644 36429112

[15] Di Cresce C , Figueredo R, Rytelewski M et al. siRNA knockdown of mitochondrial thymidine kinase 2 (TK2) sensitizes human tumor cells to gemcitabine. *Oncotarget* 2015;6:22397. doi: 10.18632/ONCOTARGET.4272 PMC4673171 26087398

[16] Grégoire V , Rosier JF, De Bast M et al. Role of deoxycytidine kinase (dCK) activity in gemcitabine’s radioenhancement in mice and human cell lines in vitro. *Radiother Oncol* 2002;63:329–38. doi: 10.1016/S0167-8140(02)00106-8 12142097

[17] Vuletić A , Mirjačić Martinović K, Spasić J. Role of histone deacetylase 6 and histone deacetylase 6 inhibition in colorectal cancer. *Pharmaceutics* 2024;16:54. doi: 10.3390/PHARMACEUTICS16010054 PMC10818982 38258065

[18] Bergman AM , Eijk PP, Ruiz Van Haperen VWT et al. In vivo induction of resistance to gemcitabine results in increased expression of ribonucleotide reductase subunit M1 as the major determinant. *Cancer Res* 2005;65:9510–16. doi: 10.1158/0008-5472.CAN-05-0989 16230416

[19] Hyang Ri K , Hyeon Gyeom C, Chae Kyung J. Butyrate-Mediated Acquisition of Chemoresistance by Human Colon Cancer Cells. *Oncol Rep* 2016;36:1119–26. 27277338 10.3892/or.2016.4838

[20] Lin JF , Hu PS, Wang YY et al. Phosphorylated NFS1 weakens oxaliplatin-based chemosensitivity of colorectal cancer by preventing PANoptosis. *Signal Transduct Target Ther* 2022;7:54. doi: 10.1038/S41392-022-00889-0 PMC8882671 35221331

[21] Merlos Rodrigo MA , Jimenez Jimemez AM, Haddad Y et al. Metallothionein isoforms as double agents – Their roles in carcinogenesis, cancer progression and chemoresistance. *Drug Resist Updat* 2020;52:100691. doi: 10.1016/J.DRUP.2020.100691 32615524

[22] William AH , Abhisek S, Kabir M. Pharmacologic Resistance in Colorectal Cancer: A Review – PMC. *Ther Adv Med Oncol* 2016;8:57–84. doi: https://doi.org/:10.1177/1758834015614530 26753006 10.1177/1758834015614530 PMC4699262

[23] Mariusz P. Pharmacogenetics Research on Chemotherapy Resistance in Colorectal Cancer Over the Last 20 Years – PMC. *World J Gastroenterol* 2014;20:9775–827. doi: 10.3748/wjg.v20.i29.9775 25110414 PMC4123365

[24] Tokunaga Y , Sasaki H, Saito T. Clinical role of orotate phosphoribosyl transferase and dihydropyrimidine dehydrogenase in colorectal cancer treated with postoperative fluoropyrimidine. *Surgery* 2007;141:346–53. doi: 10.1016/J.SURG.2006.06.025 17349846

[25] Yokogawa T , Yano W, Tsukioka S et al. dUTPase inhibition confers susceptibility to a thymidylate synthase inhibitor in DNA-repair-defective human cancer cells. *Cancer Sci* 2021;112:422–32. doi: 10.1111/cas.14718 33140501 PMC7780055

[26] Wen S , Wang X, Wang Y et al. Nucleoside diphosphate kinase 2 confers acquired 5-fluorouracil resistance in colorectal cancer cells. *Artif Cells Nanomed Biotechnol* 2018;46:896–905. doi: 10.1080/21691401.2018.1439835 29475390

[27] Li H , He J, Jia W. The influence of gut microbiota on drug metabolism and toxicity. *Expert Opin Drug Metab Toxicol* 2016;12:31. doi: 10.1517/17425255.2016.1121234 PMC5683181 26569070

[28] Kelly MG , Alvero AB, Chen R et al. TLR-4 signaling promotes tumor growth and paclitaxel chemoresistance in ovarian cancer. *Cancer Res* 2006;66:3859–68. doi: 10.1158/0008-5472.CAN-05-3948 16585214

[29] Chu H , Han N, Xu J. CMPK1 Regulated by miR-130b attenuates response to 5-FU treatment in gastric cancer. *Front Oncol* 2021;11:637470. doi: 10.3389/FONC.2021.637470/BIBTEX PMC8013733 33816278

[30] Che J , Pan L, Yang X et al. Thymidine phosphorylase expression and prognosis in colorectal cancer treated with 5-fluorouracil-based chemotherapy: a meta-analysis. *Mol Clin Oncol* 2017;7:943. doi: 10.3892/MCO.2017.1436 PMC5740914 29285354

[31] Allain EP , Rouleau M, Lévesque E et al. Emerging roles for UDP-glucuronosyltransferases in drug resistance and cancer progression. *Br J Cancer* 2020;122:1277–87. doi: 10.1038/S41416-019-0722-0 32047295 PMC7188667

[32] Yue B , Gao R, Wang Z et al. Microbiota-host-irinotecan axis: a new insight toward irinotecan chemotherapy. *Front Cell Infect Microbiol* 2021;11:710945. doi: 10.3389/FCIMB.2021.710945 PMC8553258 34722328

[33] Sharma NK , Bahot A, Sekar G et al. Understanding Cancer’s defense against topoisomerase-active drugs: a comprehensive review. *Cancers (Basel)* 2024;16:680. doi: 10.3390/CANCERS16040680 PMC10886629 38398072

[34] Ježek P . 2-Hydroxyglutarate in cancer cells. *Antioxid Redox Signal* 2020;33:903. doi: 10.1089/ARS.2019.7902 PMC7533892 31847543

[35] Hammond WA , Swaika A, Mody K. Pharmacologic resistance in colorectal cancer: a review. *Ther Adv Med Oncol* 2016;8:57. doi: 10.1177/1758834015614530 PMC4699262 26753006

[36] Ladner RD . The role of dUTPase and Uracil-DNA repair in cancer chemotherapy. *Curr Protein Pept Sci* 2005;2:361–70. doi: 10.2174/1389203013380991 12374095

[37] Steinegger M , Söding J. MMseqs2 enables sensitive protein sequence searching for the analysis of massive data sets. *Nat Biotechnol* 2017;35:1026–28. doi: 10.1038/NBT.3988 29035372

